# Evaluation of a surrogate virus neutralization assay for detecting neutralizing antibodies against SARS-CoV-2 in an African population

**DOI:** 10.1093/biomethods/bpae095

**Published:** 2024-12-23

**Authors:** Lilian Nkinda, Victoria Shayo, Salim Masoud, Godfrey Barabona, Isaac Ngare, Ponsian P Kunambi, Emmanuel Nkuwi, Doreen Kamori, Frank Msafiri, Elisha Osati, Frank Eric Hassan, Juma Kisuse, Benson Kidenya, Sayoki Mfinanga, Mbazi Senkoro, Takamasa Ueno, Eligius Lyamuya, Emmanuel Balandya

**Affiliations:** Campus College of Medicine, Muhimbili University of Health and Allied Sciences, 65001 Dar es Salaam, Tanzania; Campus College of Medicine, Muhimbili University of Health and Allied Sciences, 65001 Dar es Salaam, Tanzania; Campus College of Medicine, Muhimbili University of Health and Allied Sciences, 65001 Dar es Salaam, Tanzania; Campus College of Medicine, Muhimbili University of Health and Allied Sciences, 65001 Dar es Salaam, Tanzania; Division of Infection and Immunity, Joint Research Centre for Retrovirus Infection, Kumamoto University, 860-0811 Kumamoto, Japan; Division of Infection and Immunity, Joint Research Centre for Retrovirus Infection, Kumamoto University, 860-0811 Kumamoto, Japan; Campus College of Medicine, Muhimbili University of Health and Allied Sciences, 65001 Dar es Salaam, Tanzania; Division of Infection and Immunity, Joint Research Centre for Retrovirus Infection, Kumamoto University, 860-0811 Kumamoto, Japan; Department of Microbiology and Parasitology, University of Dodoma, 259 Dodoma, Tanzania; Campus College of Medicine, Muhimbili University of Health and Allied Sciences, 65001 Dar es Salaam, Tanzania; Division of Infection and Immunity, Joint Research Centre for Retrovirus Infection, Kumamoto University, 860-0811 Kumamoto, Japan; Campus College of Medicine, Muhimbili University of Health and Allied Sciences, 65001 Dar es Salaam, Tanzania; Management and Development for Health, 79810 Dares Salaam, Tanzania; Campus College of Medicine, Muhimbili University of Health and Allied Sciences, 65001 Dar es Salaam, Tanzania; Muhimbili National Hospital, 65000 Dar es Salaam, Tanzania; National Institution for Medical Research, Muhimbili Centre, 11101 Dar es Salaam, Tanzania; National Institution for Medical Research, Muhimbili Centre, 11101 Dar es Salaam, Tanzania; Department of Biochemistry and Molecular Biology, Catholic University of Health and Allied Sciences - Bugando, 1464 Mwanza, Tanzania; National Institution for Medical Research, Muhimbili Centre, 11101 Dar es Salaam, Tanzania; Kampala International University, Department of Public Health, Ilala 12110, Dar es Salaam, Tanzania; National Institution for Medical Research, Muhimbili Centre, 11101 Dar es Salaam, Tanzania; Campus College of Medicine, Muhimbili University of Health and Allied Sciences, 65001 Dar es Salaam, Tanzania; Division of Infection and Immunity, Joint Research Centre for Retrovirus Infection, Kumamoto University, 860-0811 Kumamoto, Japan; Campus College of Medicine, Muhimbili University of Health and Allied Sciences, 65001 Dar es Salaam, Tanzania; Campus College of Medicine, Muhimbili University of Health and Allied Sciences, 65001 Dar es Salaam, Tanzania

**Keywords:** surrogate virus neutralization assay, Tanzania, neutralizing antibodies, evaluation, SARS-CoV-2

## Abstract

The global resurgence of coronaviruses and the move to incorporate COVID-19 vaccines into the expanded program for immunization have warranted for a high-throughput and low-cost assay to measure and quantify mounted neutralizing antibodies as an indicator for protection against SARS-CoV-2. Hence, we evaluated the surrogate-virus-neutralization-assay (sVNT) as an alternative assay to the pseudo-virus neutralization assay (pVNT). The sVNT was used to measure neutralizing antibodies among 119 infected and/or vaccinated blood samples, against wild-type SARS-CoV-2 (WT) and the Omicron-variant with reference to the pVNT. Four different cut-offs were assessed for suitability in distinguishing neutralizers: the manufacturer (>30%), literature-based (>50%) and (>80%), and population-based (>27.69%). The obtained data was analyzed using “R” through its integrated development environments; JAMOV and R-Studio. Using the WT strain, only the population-based cut-off was able to differentiate neutralizers from non-neutralizers beyond chance, with an area under the curve (AUC) of 0.833 (95%CI, 0.505–1.0; *P* = .049). Applying the population-based cut-off, improved the sensitivity to 100% from 91.4% obtained from the manufacturer cut-off (*P* = .002). However, the specificity remained low (67%). The negative-predictive-value also improved to 100% vs 16.4% (*P* = .006), but there was no difference in the positive-predictive-value (99.1% vs 99.1%) (*P* = .340). When we used the Omicron-variant, the sVNT titers were not able to predict the neutralizers and non-neutralizers with reference to pVNT (AUC of 0.649) (*P* = .221). The sVNT assay is a potential alternative for screening individuals harboring potent neutralizing antibody with high sensitivity, although we recommend continuous improvement of the assay in line with the viral mutations. Further, we recommend that individual users establish a population-based cut-off while using the sVNT assay.

## Background

During the early phases of the coronavirus pandemic in 2019–2020, the World Health Organisation (WHO) named testing, isolation, and contact tracing as the backbone of the pandemic response [1]. As a result of this global move, unprecedented number serological and molecular commercial testing platforms were developed to cater for this need [2, 3], creating a demand for continued population-based evaluations before mass use.

Four years post pandemic, COVID-19 is no longer a public health emergency of international concern although the Omicron variants persist globally with increased transmissibility and enhanced immune escape [4]. Because of this the coronavirus vaccine has been added among the recommended vaccines in the expanded program for immunization globally [5]. Both the emergency of the immune escape Omicron variant and administration of COVID-19 vaccines highlighted the need to assess immune correlates necessary for continued protection, in the face of the current evolving variants and potentially another SARS-associated coronavirus pandemic or epidemic. Neutralizing antibody has been named as the immune correlates of protection against SARS-CoV-2, whereby participants with a geometrical mean concentration of 197.5 [181.9–214.4] remained uninfected with coronavirus compared to those with lower mean [6]. Hence, showing the direct presence of neutralizing antibodies is crucial for vaccine evaluation and sero-surveillance especially in sub-Saharan Africa where coronaviruses are endemic [7].

Conventional methods for functional assessment of neutralizing antibodies require challenging the patient antibodies with a replicating SARS-CoV-2 (live-virus neutralization assay) or a non-replicating virus with SARS-CoV-2 spike (pseudo-virus neutralization assay—pVNT) [8]. The ability to adopt and use these methods in Tanzania and similar settings is limited because of the cost, long assay time, and the requirement of specialized containment facilities. Alternatively, the surrogate neutralization assay is a high throughput automated enzyme linked immunosorbent assay (ELISA) with a fast turn-around time. The assay relies on competitive inhibition of neutralizing antibodies, in the interaction between the receptor binding domain (RBD) of the spike protein and angiotensin converting enzyme 2 (ACE2) cell receptors, similar to the conventional methods [8]. Hence, we evaluated the performance of the surrogate virus neutralization assay (sVNT) in detecting neutralization antibodies among convalescent and vaccinated participants with reference to the pVNT.

## Materials and methods

### Study site

Participants were recruited from the Muhimbili National Hospital, Mwananyamala, Temeke Regional Referral Hospitals, and Aga Khan Hospital in Dar-es-Salaam. Upon consent, the participants were requested to come to Muhimbili University of Health and Allied Sciences (MUHAS) in Dar-es-Salaam, Tanzania for blood collection. Plasma was separated within 30 min of collection and stored at − 80°C until time for testing. About 2–3 vials of 1.5 ml each were obtained from each participant. One vial was used for the sVNT assay at MUHAS while the other vials were transported on dry ice, maintaining a − 80°C temperature to the Joint Research Centre for Human Retroviral Infection in Kumamoto University, Japan for the pVNT assay.

### Study design

A cross-sectional study was conducted between August 2022 and January 2023.

### Study population

Blood samples from exposed adult participants (>18 years), either infected with SARS-CoV-2 virus and/or vaccinated with viral vector vaccine or the messenger ribonucleic acid (mRNA) vaccine were analyzed. During the data collection period, vaccine and infection immune responses were reported to last up to 4 and 8 months, respectively [9, 10]. Hence, all samples from the infected participants had PCR confirmation and were taken within 1–8 months post infection. Vaccinated participants had their samples taken within 1–4 months after receiving one or two doses of the initial primary vaccine series (Wuhan original Wild Type strain—WT) encompassing one dose of the viral-vector vaccine and two doses for the mRNA vaccines. Similar to previous studies [11], we assumed that the sVNT assay might be robust enough to discriminate neutralization activities between differentially exposed individuals and at different time points after exposure following infection and/or vaccination, hence the diversification of the participants. Also, analysis of the different cohorts is a reflection of the population characteristics and provided clinical relevance of the sVNT results, even in the Omicron context. Participants with immunocompromising illnesses such as HIV and malignancies were excluded from the study.

### Sample size and sampling technique

Because of high death rate among hospitalized patients upon discharge, convenient sampling method was used to recruit participants who were alive, had reachable mobile contacts and were within the proximity of the study site. A sample size of 119 was calculated using the epi R package (tools for the analysis of epidemiology data version 2.0.76) with function epi.ssdxsesp() [12]. Twenty-five participants were infected, 25 were infected-vaccinated and 69 were vaccinated individuals who had no prior symptomatic infection (self-reported). Blood samples were collected in ethylenediamine tetra acetic acid coated tubes. Plasma was separated by centrifugation and immediately stored in aliquots at − 80°C within 3 h of sample collection.

### Sample size for establishing population cut-off value in neutralizing antibodies

During the period of data collection, approximately 75% (ranging from 50% to 80%) of the population had antibody levels sufficient to neutralize the COVID-19 antigen [13–15]. The currently recommended method for screening antibody titers in the population has an area under the Receiver Operating Characteristic (ROC) curve (AUC) of 0.9713, which demonstrates diagnostic characteristics within an acceptable range [11]. Using this information, we employed the MedCalc tool (https://www.medcalc.org/) to calculate the minimum sample size required to estimate the AUC with a margin of error of 7% (0.07). The calculation yielded a required sample size of 115.

### Test and reference methods

#### Neutralization antibody analysis using the surrogate virus neutralization assay as a test method

The sVNT assay was conducted at MUHAS at biosafety level 2 laboratory. Briefly, all the plasma samples were heat-inactivated at 56°C for 45 min prior to further processing. The sVNT (Gen-Script cPass™, USA, L00847) was performed as per manufacturer’s instructions against the WT SARS-CoV-2 virus (D614G) [10] and the BA.1.1.529 omicron variant. Briefly, participants samples, and the assay’s positive and negative controls were diluted 1:10 with sample dilution buffer. The dilutions were mixed with horseradish peroxidase conjugated recombinant SARS-CoV-2 RBD solution and incubated for 30 min at 37°C. The mixtures were subsequently incubated for 15 min at 37°C in a capture plate that was pre-coated with human ACE2 protein. After a washing step, tetramethylbenzidine solution was added and the plate was incubated in the dark at room temperature for 15 min. Stop solution was added to quench the reaction and the absorbance was immediately read at 450 nm on an ELISA microplate reader. The percentage inhibition was calculated as [(1− OD value of sample/OD value of Negative control)] × 100%, where OD stands for optical density. Participants with inhibition >30% were regarded as neutralizers while those with ≤30% were regarded as non-neutralizers as per manufacturer’s instruction [10].

#### Neutralizing antibody analysis using pseudo-virus neutralization assay as a reference (gold standard) method

Briefly, human embryonic kidney 293T cells (HEK293T) were transfected with plasmids encoding ACE2/Transmembrane serine proteases (TMPRSS2) receptors using polyethyleneimine, and SARS-CoV-2 pseudo-viruses were prepared by co-transfecting the HEK293T cells with a HiBiT tagged lentiviral vector backbone and plasmids encoding the respective variants, that is, Wuhan ancestral WT lab variant—D614G and the BA.1.1.529 omicron variant were prepared as previously described [11–13]. Transfected cells were harvested after 48 h of incubation.

For neutralization assays, heat inactivated plasma (56°C for 45 min) was 5-fold serially diluted on a 96-well plate starting from a 40-fold dilution. Pseudo-viruses with SARS-CoV-2 spike proteins were thawed and added at a concentration of 6 ng of the p24 antigen per well and incubated. Next, HEK293T cells expressing ACE2/TMPRSS2 were added and incubated for 48 h before measuring the infectivity. Neutralization titer was expressed as the percent decrease in infectivity relative to that of plasma free pseudo-viruses. The 50% neutralization titer (NT_50_) value was defined as the maximum dilution required to inhibit 50% of the pseudo-virus infectivity and was determined using four-parameter nonlinear regression (GraphPad Prism). Participants with sera whose NT_50_ where 40 or below were regarded as non-neutralizers and those whose NT_50_ were above 40 were regarded as neutralizers. All sample manipulations were treated as potentially hazardous and were conducted in a biosafety level 3 laboratory.

### Statistical analysis

The obtained data was analyzed using an open source statistical programming language R (version 4.2.0) through its integrated development environments (IDEs) called JAMOV (version 2.2) and R Studio (release 2022.02.0). “DTComPair” R package version 1.1.0 was used to provide statistical test for comparing diagnostic characteristics of different cut-off values to be used in the evaluation of the sVNT assay, that is; manufacturer-recommended cut-off of 30% [10], literature-based cut-off points of 50% and 80% [14] and the population established cut-off values to be determined. The ROC curve was used first to test the extent to which results of the test assay (sVNT) can predict the results of the reference methods (pVNT), whereby AUC and a *P*-value were used to judge how best the test mirrors the reference assay. Thereafter, an optimal cut-off value that best distinguished between neutralizers and non-neutralizers beyond chance was determined using Youden’s index and a *P*-value less than 5%. An AUC of <0.5 suggested the cut-off cannot discriminate neutralizers and non-neutralizers. Explicitly, AUC of 0.5–0.6 indicates poor discrimination, 0.6–0.7 indicates sufficient discrimination, 0.7–0.8 is considered good, 0.8–0.9 is excellent, and >0.9 is outstanding [15]. Comparisons of these diagnostic characteristics of different cut-off values were performed using; McNemar test for sensitivities and specificities, and generalized score statistics was used for comparison of relative predictive positive values (PPV) and negative predictive values (NPV). Moreover, the manufacturer-recommended (30%) [10] and literature-based cut-off points 50% and 80% [14], and population established cut-off were tested prior to evaluation of the assays.

## Results

### Diagnostic metrics of the surrogate virus neutralization assay against the WT strain using the manufacturer’s cut-off

Neutralizing antibodies were tested among 119 infected and/or vaccinated participants. Their baseline characteristics were reported in our recent publication [16]. Using the manufacturer-recommended cut-off, diagnostic metrics such as sensitivity, specificity, diagnostic accuracy, and predictive capacity of the sVNT assay were evaluated with reference to the pVNT assay. As shown in Table 1, the sensitivity was high (91.4%) but the specificity was low at 66.7%, and the assay diagnostic accuracy was 90.8%. Moreover, the ability of the test to predict participants with sufficient neutralizing antibodies (neutralizers) with reference to the pseudo-virus neutralization assay (PPV) was 99.1% (almost perfect), but the ability to predictive non-neutralizers (NPV) was low at 16.7%.

**Table 1.** Diagnostic metrics of the surrogate virus neutralization assay against the WT strain using the manufacturer’s cut-off.

				95% Confidence interval			
Decision statistics		Estimate (%)		Lower (%)		Upper (%)	
Test sensitivity		91.4		84.7		95.8	
Test specificity		66.7		9.4		99.2	
Diagnostic accuracy		90.8		84.1		95.3	
Positive predictive value		99.1		94.9		100.0	
Negative predictive value		16.7		2.1		48.4	
Proportion of false positives		33.3		0.8		90.6	
Proportion of false negative		8.6		4.2		15.3	

### Establishing an optimal Cut-Off for the surrogate virus neutralization assay against the WT strain

**Figure 1.**
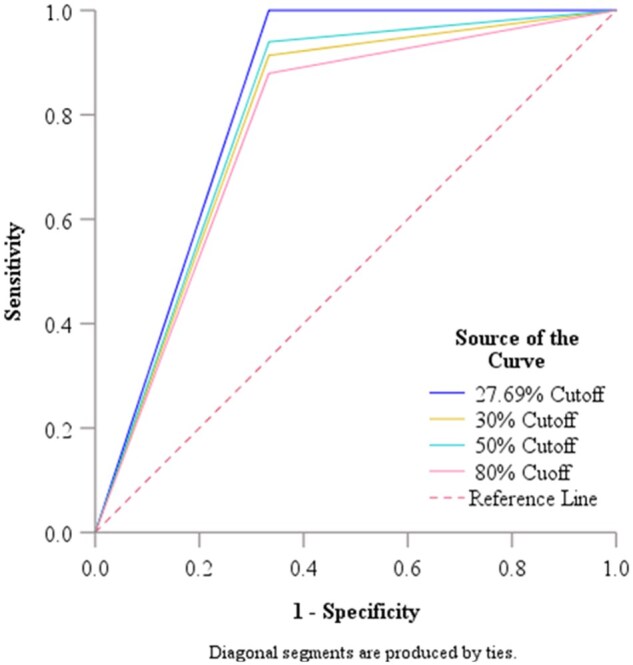
Receiver operating curve analysis for the WT strain cut-off value.

Because of the low specificity result on the sVNT assay, we used the ROC (Fig. 1) to check on whether the manufacturer-recommended cut-off, and some selected literature-based cut-off could accurately distinguish neutralizers and non-neutralizers. We got an AUC of 0.790, 95% CI (0.469–1.0), *P* = .087 for the manufacturer’s recommended cut-off (30%), AUC 0.803, 95% CI (0.480–1.0), *P* = .074 for the literature-based cut-off 50%, and AUC 0.773, 95% CI (0.454–1.0), *P* = .107, for the 80% literature-based cut-off. These results indicated good performance, although these cut-offs could not discriminate the neutralizer and non-neutralizer, beyond chance (*P* > .05), (Table 2). Hence, we decided to determine the optimal population cutoff. The calculated population-based cut-off was (27.69%) with an AUC of 0.833, 95% CI (0.505–1.0), *P* = .049, indicating excellent performance in discriminating between neutralizers and non-neutralizers when using the sVNT assay.

**Table 2.** Establishing an optimal cut-off for the surrogate virus neutralization assay against the WT strain.

			95% Confidence interval		
Variable—cutoffs	Area	Std error	Lower bound	Upper bound	*P*-value*
Population based—27.6967	0.833	0.167	0.505	1.000	**.049**
Manufacture recommended 30% Cutoff	0.790	0.164	0.469	1.000	.087
50% Cutoff	0.803	0.165	0.480	1.000	.074
80% Cutoff	0.773	0.163	0.454	1.000	.107

WT: Wild-type, Std: Standard.

* Null hypothesis: true area = 0.05.

### Evaluation of surrogate virus neutralization assay against the WT strain using the population cut-off vs the manufacturer’s cut-off value

Applying the population determined cut-off to evaluate the sVNT assay against the pVNT, significantly improved the sensitivity from 91.4% (using manufacturer’s cut-off) to 100% (*P* = .002). However, the specificity remained low at 67% similar to the manufacturer’s cut-off. Moreover, the ability of the assay to predict non-neutralizers, as participants without neutralizing antibodies (NPV), was also significantly improved from 16.4% (when manufacturer’s cut-off was used) to 100% (*P* = .006), but there was no difference in the ability to predict the neutralizers (PPV) (99.1% vs 99.1%, *P* = .340) (Table 3).

**Table 3.** Evaluation of Surrogate Virus Neutralization assay against the WT strain using the population cut-off vs the manufacturer’s cut-off value.

	WT cutoff value, CI				
Diagnostic metrics	27.69%	30.0%	Difference	Test statistics	*P*-value
Sensitivity	100.0% (96.9%–100%)	91.4% (84.7%–95.8%)	−8.6	10.00	.002
Specificity	66.7% (9.4%–99.2%)	66.7% (9.4%–99.2%)	0.0	NA	NA
PPV	99.1% (95.3%–100%)	99.1% (94.9%–100%)	0.999	−0.953	.340
NPV	100.0% (15.8%–100%)	16.7% (2.1%–48.4%)	0.167	−2.776	.006

PPV: Positive predictive value, NPV: Negative predictive value, NA: Not applicable.

### Evaluation of surrogate virus neutralization assay against the omicron variant with reference to the pseudo-virus neutralization assay

Moreover, we analyzed the sVNT omicron antibody titers readings with reference to the pVNT readings **(**Fig. 2**)**. First, we begun by scrutinizing if the sVNT antibody titers were able to predict neutralizers vs non-neutralizer on the reference method, before establishing the population cut-off. We found that the sVNT results were not able to predict neutralizers and non-neutralizers beyond chance, with an AUC of 0.649 and a *P*-value of .221 (Table 4). Hence, further analysis using the cut-offs was unnecessary.

**Figure:**
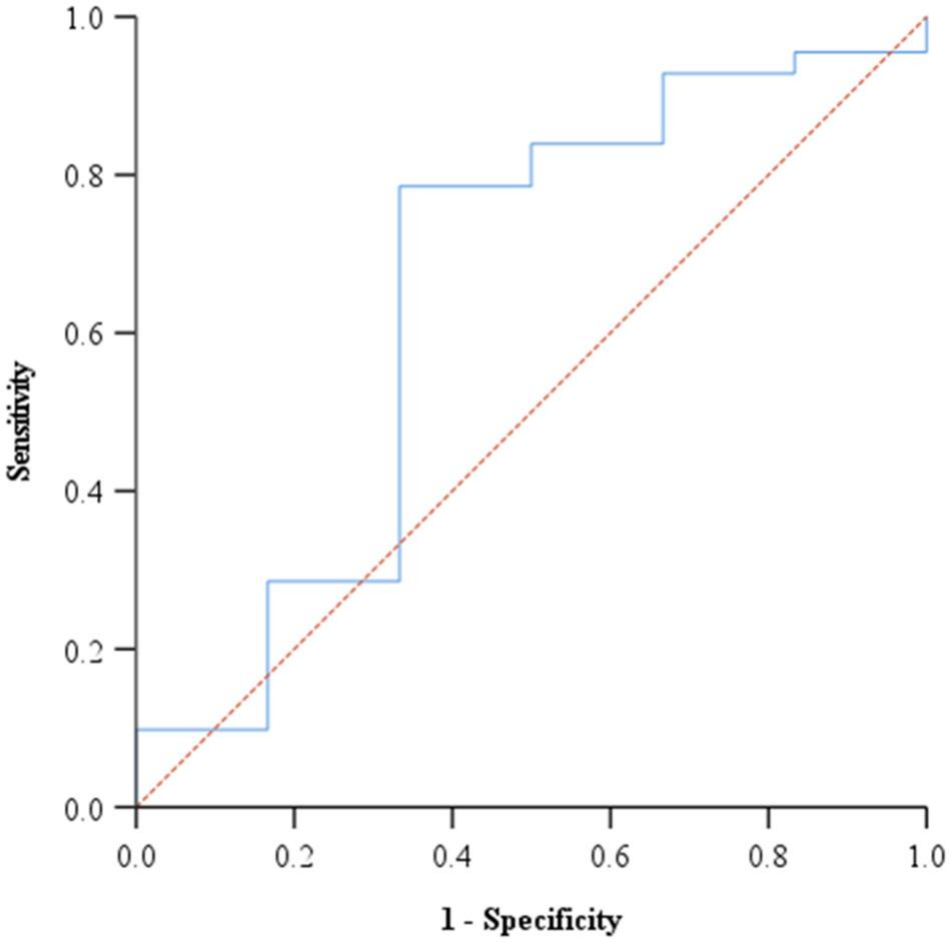
Figure 2. Receiver operating curve analysis for the Omicron variant cut-off value.

**Table 4.** Evaluation of surrogate virus neutralization assay against the omicron variant with reference to the pseudo-virus neutralization assay.

Area	Std error	95% Confidence interval		
		Lower bound	Upper bound	*P*-value*
0.649	0.138	0.379	0.919	.221

* Null hypothesis: true area = 0.05.

## Discussion

To overcome challenges presented by conventional methods of analyzing functional neutralizing antibodies against SARS-CoV-2 in Tanzania, this study evaluated the sVNT assay as a low-cost alternative with high throughput and a short turnaround time to distinguish neutralizers and non-neutralizers among exposed individuals with reference to the pVNT assay. Using the WT strain, we show that the sVNT assay had the sensitivity of 100%, diagnostic accuracy of 99%, NPV of 100%, a PPV of 99%, and specificity of 66.7%. Notwithstanding the low specificity, our findings indicate that the sVNT has potential to be used as an alternative tool for population-based sero-surveillance of SARS-CoV-2, particularly in settings were the adoption of pVNT assays will take time.

Our study aimed to investigate whether the surrogate neutralizing assay could be used as a high throughput assay to screen for neutralizing antibodies and distinguish neutralizers and non-neutralizers with reference to the pseudo-virus based assay. Hence, we tested the manufacturer-recommended cut-off and the literature-based cut-off, which unfortunately showed incapacity to distinguish neutralizers from non-neutralizers beyond chance. Upon applying the population-based cut-off, the sensitivity was significantly improved to 100% from 91% of the manufacturer cut-off (*P* = .002), 94%, and 87.9%, of the 50% (*P* = .008), and 80% (*P* = .001) literature-based cut-off, respectively. There was no difference on the specificity and PPV, for all cut-offs, however, the NPV, significantly improved from 16.7% to 100%, 22% to 100%, 12.5% to 100% for the manufacturer cut-off, and the 50% and 80% literature-based cut-off, respectively. Although the ROC determined population cut-off differ for every population depending on ethnicity, geographical background, and prevalence of SARS-CoV-2, still similar outcomes where observed; overall there was improvement in the diagnostic performance to the reference method [17, 18]. Hence, this assay may require specific cut-off with respect to the population it will be applied for [18, 19]. Therefore, moving forward, we evaluated the surrogate neutralizing assay with reference to the pseudoviral neutralization using the population based cut off of 27.69%.

The comparison of the surrogate virus neutralization assay and the pseudoviral neutralization assay showed a sensitivity of 100%. This is consistent to studies reported elsewhere, whereby the sensitivity tended to range between 90% and 100% [18–21], even when the manufacturer cut-off was used [8]. This could be attributed by the ability of the assay to detect total neutralizing antibodies in an isotype independent manner hence improving the sensitivity [8, 18]. The low sensitivity in some of the reports was attributed by the presence of non-RBD neutralizing antibodies, of which the surrogate assay has limited capacity to identify, especially when the tested population is predominantly unvaccinated [18], unlike in the current study.

In our study, the sVNT assay classified 33% of individuals without antibodies as having neutralizing antibodies. This resulted in a high rate of false positives, as reflected in its low specificity of 66.7%. Although we had limited number of non-neutralizing participants in the current study, similar results were obtained by Gillot *et al.*, who reported a specificity of 66.67% when pre-pandemic samples were tested [19]. Similarly, Silva *et al.* demonstrated an impressive sensitivity of 98.7% but a poor specificity of 63.2% [11]. Findings from Adams *et al*. reported that the sVNT assay exhibits low specificity, particularly among convalescent (28%) relative to vaccinated individuals (50%) [22]. In order to further elucidate the low specificity of sVNT, a recent systematic review showed that the sVNT tends to incorrectly identify 34%–58% of non-neutralizers as neutralizers [23–25]. The inability of the sVNT to discriminate between the mere presence versus actual functional capacity to inhibit viral entry into host cells may be the main source of the low specificity and hence limitation of the sVNT [17]. As a remedy, Bond *et al.,* suggested using a higher inhibition cut-off to improve specificity [17]. Kweon *et al.* estimated a cut off of 83% using the ROC curve, and the specificity was 97.8% [21]. In the current study, we explored cut-offs ranging from 27% to 80%. However, specificity was not improved. Alternatively, introducing an equivocal range for the assay, determined through repeated testing, might help mitigate these variations [17, 18]. Future studies should explore further ways of optimizing the specificity of the sVNT to improve on its utility.

Moreover, our study also evaluated the utility and adaptability of the sVNT assay in diagnosis SARS-CoV-2 omicron variants. Previous findings reported the sVNT to fair moderately well with respect to the viral mutations from the WT to Omicron subvariants that is BA1, BA2, BA 5, with sensitivity of 100% (WT) and 57%–100% (BA1, BA2, BA 5) [11]. In the current study, analysis of the Omicron variant on the ROC showed inability of sVNT to predict those of the reference assay (AUC of 0.649, *P* = 0.22). According to Silva *et al.*, the sVNT has limited capacity of detecting low to intermediate neutralization titers, displaying very poor sensitivity of 69% and 42% for BA2 and BA5, respectively, using the manufacturer’s cut-off [11]. When analysis was confined to a 3-dose vaccine-boosted population with higher antibody titers, the sensitivity of BA2 and BA.5 improved to 100% and 73.9%, respectively [11]. Our study is concordant with this observation as we reported an AUC of 0.728, *P* = .0001, when titers below 250 are excluded (Supplementary material). Although it is not clear to what extent antibody just above the detection point are protective against symptomatic infection, the reference method counts antibody titers greater than 40 neutralization titers to have ability to inhibit 50% of the viral infectivity *in vitro*. This finding highlights the need for continued improvement of the assay, as antibody responses are known to wane overtime, and with low vaccination rates in African settings, the antibody titers are likely to stay at modest to low levels [22].

Our study has several limitations. First we did not conduct multiple repeated testing to establish an equivocal range that can be used to mitigate the assay variations. Also, we did not have pre-pandemic samples; hence, samples that tested negative on the reference method were considered negative.

In conclusion, the surrogate virus neutralization assay is a viable alternative to live virus and pseudo-virus neutralization assays with a great potential for utility in population-based sero-surveillance for SARS-CoV-2 in low-resource settings. However, there is need for continuous improvement of the assay to enhance its diagnostic capacity, especially in the face of ongoing viral mutations.

## Supplementary Material

bpae095_Supplementary_Data

## Data Availability

Data used to draw this conclusion are available from the corresponding author on reasonable request.

## References

[1] Peeling RW , Heymann DL, Teo Y-Y et al Diagnostics for COVID-19: moving from pandemic response to control. The Lancet 2022;399:757–68. 10.1016/S0140-6736(21)02346-1 PMC8687671 34942102

[2] Chavda V , Valu D, Parikh P et al Conventional and novel diagnostic tools for the diagnosis of emerging SARS-CoV-2 variants. Vaccines 2023;11:374. 10.3390/vaccines11020374 36851252 PMC9960989

[3] Ghaffari A , Meurant R, Ardakani ALI. COVID-19 point-of-care diagnostics that satisfy global target product profiles. Diagnostics 2021;11:115. 10.3390/diagnostics11010115 33445727 PMC7828180

[4] Burki T. WHO ends the COVID-19 public health emergency. Lancet Respir Med 2023;11:588. 10.1016/S2213-2600(23)00217-5 37247628 PMC10219632

[5] World Health Organisation 2023. COVID-19 advice for the public: Getting vaccinated. https://www.who.int/emergencies/diseases/novel-coronavirus-2019/covid-19-vaccines/advice (5 August 2024, date last accessed).

[6] Regev-Yochay G , Lustig Y, Joseph G et al Correlates of protection against COVID-19 infection and intensity of symptomatic disease in vaccinated individuals exposed to SARS-CoV-2 in households in Israel (ICoFS): a prospective cohort study. Lancet Microbe 2023;4:e309–e318. 10.1016/S2666-5247(23)00012-5 36963419 PMC10030121

[7] Tambe LAM , Mathobo P, Munzhedzi M et al Prevalence and molecular epidemiology of human coronaviruses in Africa prior to the SARS-CoV-2 outbreak: a systematic review. Viruses 2023;15:2146. 10.3390/v15112146 38005824 PMC10675249

[8] Tan CW , Chia WN, Qin X et al A SARS-CoV-2 surrogate virus neutralization test based on antibody-mediated blockage of ACE2–spike protein–protein interaction. Nat Biotechnol 2020;38:1073–8. 10.1038/s41587-020-0631-z 32704169

[9] Ssentongo P , Ssentongo AE, Voleti N et al SARS-CoV-2 vaccine effectiveness against infection, symptomatic and severe COVID-19: a systematic review and meta-analysis. BMC Infect Dis 2022;22:439. 10.1186/s12879-022-07418-y 35525973 PMC9077344

[10] Dan JM , Mateus J, Kato Y et al Immunological memory to SARS-CoV-2 assessed for up to 8 months after infection. Science 2021;371. 10.1126/science.abf4063 PMC7919858 33408181

[11] Santos da Silva E , Servais J-Y, Kohnen M et al Validation of a SARS-CoV-2 surrogate neutralization test detecting neutralizing antibodies against the major variants of concern. Int J Mol Sci 2023;24. 10.3390/ijms241914965 PMC10573711 37834413

[12] Hajian-Tilaki K. Sample size estimation in diagnostic test studies of biomedical informatics. J Biomed Inform 2014;48:193–204. 10.1016/j.jbi.2014.02.013 24582925

[13] Lyimo E , Fougeroux C, Malabeja A et al Seroprevalence of SARS-CoV-2 antibodies among children and adolescents recruited in a malariometric survey in north-eastern Tanzania July 2021. BMC Infect Dis 2022;22:846. 10.1186/s12879-022-07820-6 36371172 PMC9652923

[14] Nyawale HA , Moremi N, Mohamed M et al High seroprevalence of SARS-CoV-2 in Mwanza, northwestern Tanzania: a population-based survey. IJERPH 2022;19:11664. 10.3390/ijerph191811664 36141938 PMC9517516

[15] Salum SS , Sheikh MA, Hebestreit A et al Anti SARS-cov-2 seroprevalence in Zanzibar in 2021 before the omicron wave. IJID Reg 2022;4:120–2. 35814620 10.1016/j.ijregi.2022.06.007 PMC9251954

[16] Nkinda L , Barabona G, Ngare I et al Evaluation of cross-neutralizing immunity following COVID-19 primary series vaccination during the Omicron surge in Tanzania. J Med Virol 2024;96:e29822. 10.1002/jmv.29822 39056238

[17] Bond K , Nicholson S, Lim SM et al Evaluation of serological tests for SARS-CoV-2: implications for serology testing in a low-prevalence setting. J Infect Dis 2020;222:1280–8. 10.1093/infdis/jiaa467 32761124 PMC7454699

[18] Mahmoud SA , Ganesan S, Naik S et al Serological assays for assessing postvaccination SARS-CoV-2 antibody response. Microbiol Spectr 2021;9:e0073321. 10.1128/spectrum.00733-21 34585943 PMC8557923

[19] Gillot C , Favresse J, David C et al An evaluation of a SARS-CoV-2 pseudovirus neutralization test and a comparison to a SARS-CoV-2 surrogate virus neutralization test in a COVID-19 long-term follow-up cohort. Microbiol Res 2024;15:422–30. 10.3390/microbiolres15010028

[20] Schoefbaenker M , Neddermeyer R, Guenther T et al Surrogate virus neutralisation test based on nanoluciferase-tagged antigens to quantify inhibitory antibodies against SARS-CoV-2 and characterise omicron-specific reactivity in a vaccination cohort. Vaccines 2023;11:1832. 10.3390/vaccines11121832 38140236 PMC10748151

[21] Kweon OJ , Bae J-Y, Lim YK et al Performance evaluation of newly developed surrogate virus neutralization tests for detecting neutralizing antibodies against SARS-CoV-2. Sci Rep 2023;13:4961. 10.1038/s41598-023-31114-9 36973368 PMC10041486

[22] Adams O , Andrée M, Hermsen D et al Comparison of commercial SARS-CoV-2 surrogate neutralization assays with a full virus endpoint dilution neutralization test in two different cohorts. J Virol Methods 2022;307:114569. 10.1016/j.jviromet.2022.114569 35724697 PMC9212436

[23] Rogers TF , Zhao F, Huang D et al Isolation of potent SARS-CoV-2 neutralizing antibodies and protection from disease in a small animal model. Science 2020;369:956–63. 10.1126/science.abc7520 32540903 32540903 PMC7299280

[24] Alsoussi WB , Turner JS, Case JB et al A potently neutralizing antibody protects mice against SARS-CoV-2 infection. J Immunol 2020;205:915–22. 10.4049/jimmunol.2000583 32591393 PMC7566074

[25] Valcourt EJ , Manguiat K, Robinson A et al Evaluation of a commercially-available surrogate virus neutralization test for severe acute respiratory syndrome coronavirus-2 (SARS-CoV-2). Diagn Microbiol Infect Dis 2021;99:115294. 10.1016/j.diagmicrobio.2020.115294 33387896 PMC7758721

