# Supplementary material for: Evaluation of a surrogate virus neutralization assay for detecting neutralizing antibodies against SARS-CoV-2 in an African population
Source: Biol Methods Protoc. 2024 Dec 23;10(1):bpae095. doi: 10.1093/biomethods/bpae095 (PMC11769676; doi:10.1093/biomethods/bpae095)
Supplement: bpae095_Supplementary_Data [file bpae095_supplementary_data.zip › 1aac5_1.Supplementary document_ analysis of Omicron variant excluding low-intermidiate titres.docx]

**Evaluation of Surrogate Virus Neutralization assay against the Omicron variant with reference to the pseudo-virus Neutralization Assay, when antibody titres <250 are excluded.**


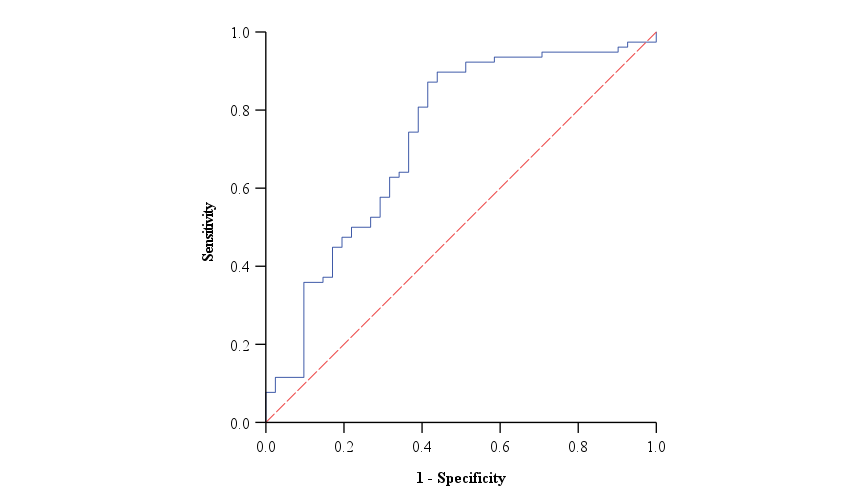


**Figure S:** Receiver operating curve analysis for the Omicron variant cut-off value, with antibody titres above 250

**Table S: Evaluation of Surrogate Virus Neutralization assay against the Omicron variant with reference to the pseudo-virus Neutralization Assay, when antibody titres <250 are excluded.**

The sVNT results for the Omicron variant were not able to predict neutralizers and non-neutralizers beyond chance, with reference to the pVNT, with AUC of 0.649 and a p-value of 0.221 on **Table 04 (main document)**. Previous literature, reported, limited capacity of the assay to detect low-intermediate (40-250) antibody tires as one of the assay’s limitations, hence 44/119 participants were excluded from this analysis on the ROC curve (**Figure S**). On **Table S**, we report an AUC of 0.728, p=0.0001 denoting good capacity in discriminating neutralizers and non-neutralizers beyond chance when high antibody titers (>250) were analyzed.

| **Area Under the Curve** | | | | |
| --- | --- | --- | --- | --- |
| Area | Std. Error^a^ | Asymptotic Sig.^b^ | Asymptotic 95% Confidence Interval | |
|  |  |  | Lower Bound | Upper Bound |
| .728 | .052 | .000 | .627 | .829 |
| a. Under the nonparametric assumption | | | | |
| b. Null hypothesis: true area = 0.5 | | | | |
